# Expression of Drosophila Matrix Metalloproteinases in Cultured Cell Lines Alters Neural and Glial Cell Morphology

**DOI:** 10.3389/fcell.2021.610887

**Published:** 2021-05-13

**Authors:** Scoty Hearst, Andrea Bednářová, Benjamin Draughn, Kennadi Johnson, Desiree Mills, Cendonia Thomas, Jendaya Scales, Eadie T. Keenan, Jewellian V. Welcher, Natraj Krishnan

**Affiliations:** ^1^Department of Biology, Tougaloo College, Tougaloo, MS, United States; ^2^Department of Chemistry and Biochemistry, Mississippi College, Clinton, MS, United States; ^3^Department of Biochemistry and Physiology, Institute of Entomology, Biology Centre, Czech Academy of Sciences, České Budějovice, Czechia; ^4^Department of Biochemistry, Molecular Biology, Entomology and Plant Pathology, Mississippi State University, Starkville, MS, United States

**Keywords:** *Drosophila melanogaster*, matrix metalloproteinases, SH-SY5Y neuroblastoma, C6 glioblastoma, cell line, apoptosis, differentiation

## Abstract

Matrix metalloproteinases (MMPs) are zinc- and calcium- dependent endopeptidases that play pivotal roles in many biological processes. The expression of several MMPs in the central nervous system (CNS) have been shown to change in response to injury and various neurological/neurodegenerative disorders. While extracellular MMPs degrade the extracellular matrix (ECM) and regulate cell surface receptor signaling, the intracellular functions of MMPs or their roles in CNS disorders is unclear. Around 23 different MMPs are found in the human genome with overlapping function, making analysis of the intracellular role of human MMPs a daunting task. However, the fruit fly *Drosophila melanogaster* genome encodes only two MMPs: dMMP1 and dMMP2. To better understand the intracellular role of MMPs in the CNS, we expressed Green Fluorescent Protein (GFP)- tagged dMMPs in SH-SY5Y neuroblastoma cells and C6 glioblastoma cell lines. Lipofection of GFP-dMMPs in SH-SY5Y cells enhanced nuclear rupture and reduced cell viability (coupled with increased apoptosis) as compared to GFP alone. In non-liposomal transfection experiments, dMMP1 localizes to both the cytoplasm and the nucleus whereas dMMP2 had predominantly cytoplasmic localization in both neural and glial cell lines. Cytoplasmic localization demonstrated co-localization of dMMPs with cytoskeleton proteins which suggests a possible role of dMMPs in cell morphology. This was further supported by transient dMMP expression experiments that showed that dMMPs significantly increased neurite formation and length in neuronal cell lines. Inhibition of endogenous MMPs decreased neurite formation, length and βIII Tubulin protein levels in differentiated SH-SY5Y cells. Further, transient expression experiments showed similar changes in glial cell morphology, wherein dMMP expression increased glial process formation and process length. Interestingly, C6 cells expressing dMMPs had a glia-like appearance, suggesting MMPs may be involved in intracellular glial differentiation. Inhibition or suppression of endogenous MMPs in C6 cells increased process formation, increased process length, modulated GFAP protein expression, and induced distinct glial-like phenotypes. Taken together, our results strongly support the intracellular role that dMMPs can play in apoptosis, cytoskeleton remodeling, and cell differentiation. Our studies further reinforce the use of Drosophila MMPs to dissect out the precise mechanisms whereby they exert their intracellular roles in CNS disorders.

## Introduction

Matrix metalloproteases (MMPs) are a family of zinc- and calcium-dependent endopeptidases that were originally characterized as secreted proteases responsible for degrading extracellular matrix (ECM) proteins. The ECM is a complex and dynamic facet of tissue architecture which is known to play fundamental roles in development, wound healing, tissue homeostasis and a host of pathological processes (De Simone and Mecham, 2013). MMPs are the most well-known effectors of ECM remodeling and play a pivotal role in many biological processes such as: cell migration, differentiation, proliferation, cell survival, embryonic development, morphogenesis, reproduction, and tissue remodeling (Nagase et al., 2006; Barkho et al., 2008; Murphy, 2011; Kessenbrock et al., 2013, 2015; Brkic et al., 2015a; Klein et al., 2015). Dysregulation of MMPs has been implicated to be the proximal factor in many diseases and disorders such as: cancer, neurodegenerative disease, arthritis, cardiovascular diseases, and fibrotic disorders (Martel-Pelletier et al., 1994; Wang et al., 2002; Craig et al., 2015; Radisky et al., 2017; Rivera et al., 2019). MMPs are zymogens, requiring proteolytic cleavage to achieve activation; humans express 23 different MMPs regulated by four different types of tissue inhibitors of metalloproteinases (TIMPs) (Bodden et al., 1994; Nagase et al., 2006; Murphy, 2011; Lukaszewicz-Zajac et al., 2014).

In principle, MMPs are essential in intracellular as well as ECM remodeling (Sinha et al., 2014). Much is known about the extracellular roles of MMPs. As mentioned before, MMPs can degrade the ECM, regulate cell surface proteins, and mediate signal transduction by degrading cell surface receptors and signaling molecules (Bodden et al., 1994; Nagase et al., 2006; Murphy, 2011; Lukaszewicz-Zajac et al., 2014). MMPs are present in many cells of the central nervous system (CNS), where extracellular MMPs are thought to play a pathological role in the neurodegenerative diseases by mediating neuroinflammation, blood-brain barrier (BBB) disruption, synaptic dysfunction, or neuronal death (Rosenberg, 2009; Horstmann et al., 2010; Miller et al., 2010; Rivera et al., 2019). Increased expression of MMPs has been reported in neurodegenerative diseases such as traumatic brain injury, stroke, meningitis, spinal cord injury, amyotrophic lateral sclerosis, Huntington’s disease, multiple sclerosis, Alzheimer’s disease, and Parkinson’s disease (Leppert et al., 1998; Lorenzl et al., 2004; Miller et al., 2010; Annese et al., 2014; Kaplan et al., 2014; Lukaszewicz-Zajac et al., 2014; Brkic et al., 2015b; Abdul-Muneer et al., 2017). MMPs are secreted by microglia, astrocytes, and neurons; and under normal physiological conditions, MMPs are present at undetectable levels in the mature brain (Brkic et al., 2015a). During neuroinflammation, extracellular MMPs are thought to breakdown the BBB by degrading the ECM and destroying tight junctions, which allows invasion of immune cells into the brain and reactive astrocytic cells into nerve tissue causing neuronal cell death (Rosenberg, 2009). Dysregulation of extracellular MMPs can be detrimental to neuronal function and enhanced neurodegeneration; however, little is known of the intracellular roles of MMPs, nor their function in CNS disorders.

It has been hypothesized that MMPs may play an important role in cytoskeleton remodeling; however, such evidence is scarce or even lacking (Bildyug, 2016). A major challenge to studying MMP function in the nervous systems of mammalian systems is that there is a general trend of expansion and specialization in MMPs with metazoan evolution due to which mammals have roughly two dozen MMP orthologs [24 in mice (*Mus musculus*) and 23 in humans (*Homo sapiens*)] and other vertebrate models generally have comparable numbers [25 in zebrafish (*Danio rerio*), 26 in the African clawed frog (*Xenopus laevis*)] with redundancy in certain functions (Jackson et al., 2010). Hence, studying MMP biology in model organisms with simple MMP families allows for experiments that elucidate the function of specific proteases. The fruit fly *Drosophila melanogaster* with only two MMP genes, dMMP1 and dMMP2 and only one tissue inhibitor of metalloproteinase (TIMP), dTIMP1 (Page-McCaw et al., 2003; LaFever et al., 2017) offers an excellent model system to investigate MMP function in the nervous system. In the fly, MMP activity in the developing nervous system is essential for both axon pathfinding and dendritic plasticity in the brain (Kuo et al., 2005). While the power of studying the simple MMP/TIMP system of the fly is obvious, it makes it difficult to generalize these functions by homologous MMPs in vertebrates and specifically humans difficult. To circumvent this, an *in vitro* approach to express dMMPs in mammalian cell lines to reveal biologically relevant activities of MMP orthologs in the nervous system is desirable. Hence, to generate a robust *in vitro* system to elucidate the intracellular function of MMPs in nervous system, we decided to express dMMPs in human neuronal and rat glial cell lines. This study lays the foundation for further research on unraveling the discrete molecular mechanisms underlying the intracellular role of MMPs in altering neural and glial cell morphology.

## Materials and Methods

### Sequence Comparison and Phylogenetic Analysis of Drosophila Matrix Metalloproteinases dMMP1 and dMMP2 With Human MMPs and NLS Prediction

The full-length amino acid sequence alignments of dMMP1 and dMMP2 with the full-length amino acid sequence of 23 human MMPs ranging from MMP1 to MMP28 was conducted using Clustal Omega^1^ (Sievers and Higgins, 2018; Madeira et al., 2019) with default settings. To generate alignments of specific dMMPs with hMMPs or rMMPs (from *Rattus norvegicus*), the TCoffee server was utilized^2^ with FASTA alignments being exported to BoxShade server^3^ for generating the alignment images. A phylogenetic tree was constructed with MEGA 7.0 using the neighbor-joining (NJ) method. The reliability of the NJ tree topology was tested by bootstrap analysis with 1000 replicates. Nuclear localization signal sequences were predicted for dMMP1 and dMMP2 using NLS Mapper^4^ (Kosugi et al., 2009).

### Generation of GFP-dMMP Constructs

Recombinant plasmids for expression of dMMPs were constructed by GenScript cloning services (GenScript, Piscataway, NJ, United States) using standard protocols by insertion of ORF of dMMP with or without the propeptide domain into vector pcDNA3.1(+)-N-eGFP resulting in GFP-dMMP expression under the CMV promoter (Supplementary Figure 1). We constructed GFP tagged dMMP1 and dMMP2 full length proteins as well as the active protease with the auto-inhibitory propeptide domain removed, denoted as dMMP1ΔPP and dMMP2ΔPP prepared for mammalian expression in the pcDNA3.1(+)-N-eGFP vector (Supplementary Figure 1). GFP-dMMP plasmids were analyzed for accuracy using DNA sequencing and restriction digest. For a GFP expression control, we used the empty pcDNA3.1(+)-N-eGFP vector (Genscript) to express the GFP protein alone. This technique has been successfully used in studying human MMP3 in previous reports (Si-Tayeb et al., 2006).

### Cell Culture and GFP-dMMP Expression

Human SH-SY5Y neuroblastoma and rat C6 glioblastoma cell lines were obtained from the American Type Culture Collection (ATCC, Manassas, VA, United States). SH-SY5Y cells were maintained in DMEM media (Fisher, Houston, TX, United States) supplemented with 15% FBS, penicillin-streptomycin (Sigma-Aldrich, St. Louis, MO, United States) and grown in an incubator at 37°C in the presence of 5% CO_2_ (Hearst et al., 2011). C6 cells were maintained in DMEM media (Fisher, Houston, TX, United States) supplemented with 10% FBS, penicillin-streptomycin (Sigma), and grown in an incubator at 37°C in the presence of 5% CO_2_. GFP control or dMMP plasmid DNA was transfected into cell lines using either Lipofectamine 2000 (Invitrogen, Carlsbad, CA, United States), or FuGene 6 (Roche, Indianapolis, IN, United States) according to the manufacturer’s instructions. SH-SY5Y stable cell lines were produced by transfecting with GFP using Fugene 6 transfection reagent and selected by G418 (G418 sulfate 600 μg/ml, Sigma) resistance as previously described (Hearst et al., 2011). Cell viability post transfection was assessed using the MTT assay using methods as previously described (Datki et al., 2003). All experiments were conducted with three independent biological repeats.

### Neurite and Glial Process Measurements

Cells were subjected to treatment with 25 nM of 12-*O*-tetradecanoylphorbol-13-acetate (TPA), MMP inhibitor III 1.5 to 100 μM, doxycycline 10 μg/ml to 40 μg/ml, or 12 μM forskolin (Sigma) for 3 days to produce differentiated cell lines as described previously (Hearst et al., 2011). Cells for immunofluorescence experiments were placed on slide chambers, fixed and probed with the primary GFP, βIII Tubulin, or GFAP and fluorescent secondary antibodies Alexa 488 and Alexa 546 (Invitrogen) followed by DAPI (Invitrogen) staining as described previously (Hearst et al., 2011). Cells were observed on an Olympus BX60 epifluorescence microscope using 40x or 100x objectives (Spectra Services, Ontario, NY, United States). Digital images were taken using the QIClick Color camera with Q capture software (Spectra Services). Changes in neurite length or process length were measured using Image J software^5^, a public domain Java image processing program provided by the Research Services Branch, National Institute of Mental Health, Bethesda, MD, United States, as previously described (Hearst et al., 2011). Each experiment was repeated thrice independently. Images of glia under various conditions were converted to binary images and then edges were found to create an edged image using ImageJ software from methods as previously described (Hearst et al., 2014).

### Western Blotting

For western blotting, cell lysates were generated by resuspending cells in RIPA buffer (Sigma), then sonicated as previously described (Hearst et al., 2010). Lysates were subjected to sodium dodecyl sulfate– polyacrylamide gel electrophoresis (4–20% acrylamide precast gels; Bio-Rad Laboratories, Hercules, CA, United States) as described earlier (Hearst et al., 2010). Equal amounts of proteins were loaded in each well. Proteins were transferred to polyvinylidene difluoride membrane (Bio-Rad), blocked for 1 h with blocking solution (1X TBS, 0.1% Tween-20 with 5% w/v non-fat dry milk) and incubated overnight with primary antibodies: Casp-3 and βIII Tubulin (Santa Cruz Biotechnology, Santa Cruz, CA, United States), GFAP and ACTB (Cell Signaling Technology, Danvers, MA, United States). Western blots were imaged using Alexa-794 secondary antibodies (Santa Cruz) and the Odyssey Infrared Imaging System (LI-COR Biosciences, NE, United States). Western blots were analyzed using Image J software, as previously described (Hearst et al., 2010). The average ratios from protein band optical densities were obtained from three independent experimental repeats.

### Statistical Analysis

Data from three independent experiments were compiled and subjected to statistical analysis. Data are expressed as Mean ± SEM or SD (when mean of ratios is represented). Statistical significance was calculated using the One-Way ANOVA with Barlett’s test for equal variances and Bonferroni’s *post hoc* test, where *p* < 0.05 was considered significant using GraphPad Prism v 6.0 (San Diego, CA, United States).

## Results

### Amino Acid Sequence Alignment Reveals dMMP–hMMP Protein Homology

**FIGURE 1:**
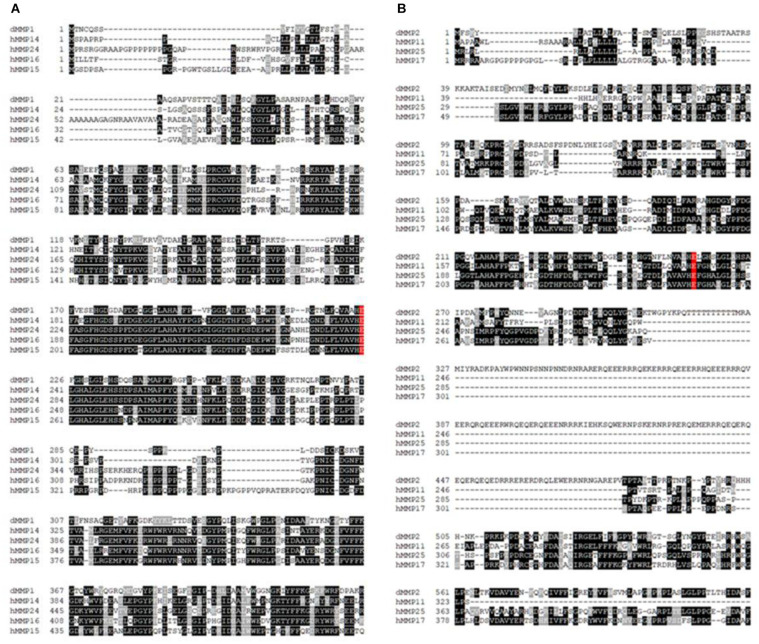
Amino acid sequence alignments of **(A)** dMMP1 and **(B)** dMMP2 with human homologs. Identical amino acids are shown in black boxes and similar amino acids are highlighted in gray boxes. Gaps have been introduced to permit alignment. Conserved active sites are shaded in red.

The protein sequence homology between dMMPs and the 23 known hMMPs was conducted using amino acid sequence alignment. dMMP1 was found to be homologous to hMMPs: 14, 15, 16, and 24 (Figure 1A); and also homologous to *R. norvegicus* MMP 14, 16, and 24 (Supplementary Figure 2A). Alignment of dMMP1 with hMMPs showed the followed sequence identities: hMMP14- 38.2%, hMMP24- 37.6%, hMMP16- 36.8%, and hMMP15-34.9%. The active sites (shaded in red) were all identical across all sequences compared (Figure 1A). Also, alignment of dMMP1 with rMMPs showed the following sequence identities: rMMP14- 38.4%, rMMP16-36.8% and rMMP24-37.7% (Supplementary Figure 2A). dMMP2 was homologous with hMMPs: 11, 17, and 25 (Figure 1B) and to rMMP 8, 14, and 16 (Supplementary Figure 2B). In case of dMMP2, hMMP11 showed 40.6%, hMMP25-39.6% and hMMP17-38.1% sequence identity respectively with all active sites (shaded in red) identical in all sequences compared (Figure 1B). Similarly, alignment of dMMP2 with rMMP showed: rMMP8-37.5%, rMMP14- 40.2% and rMMP16 40.3% sequence similarity (Supplementary Figure 2B). For a simple sequence comparison, homology results were used to construct a phylogenetic tree (Supplementary Figures 3A,B). Taken together, this homology data suggests that hMMPs, rMMPs, and dMMPs may have similar protein structure and overlapping cellular function.

### Predicted Presence of Nuclear Localization Signal Sequence in dMMP

Presence of three putative nuclear localization signal/sequences (NLS) was predicted using NLS Mapper in dMMP1 whereas a single NLS was predicted in dMMP2 (Supplementary Figures 4A,B). In both cases the entire region of the protein (amino acid sequence) was scanned.

### Lipofection of dMMPs in SH-SY5Y Neuronal Cells Results in Decreased Cell Viability Indicative of Apoptosis

**FIGURE 2:**
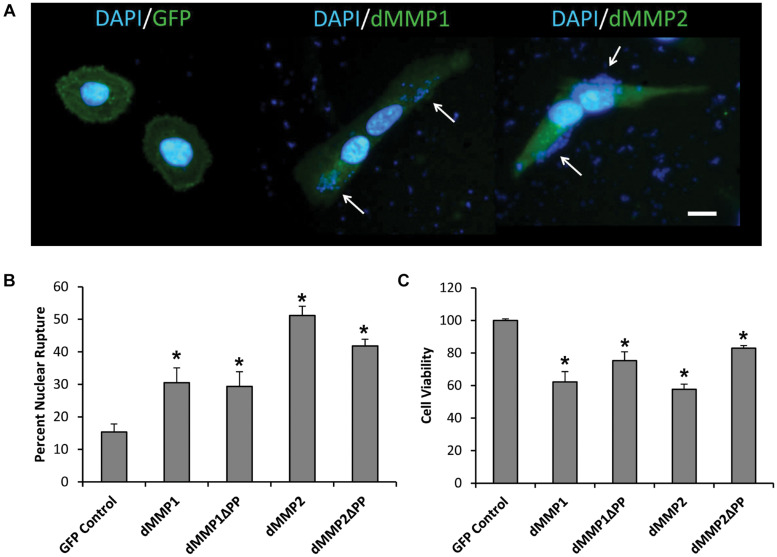
dMMPs expression promotes nuclear rupture and reduces cell viability in neuronal cells. **(A)** SH-SY5Y cells lipofected with GFP, GFP-dMMP1, and GFP-dMMP2 shown in green and DAPI nuclear stain shown in blue. Arrows point to nuclear rupture. Scale bar is 10 μm. **(B)** Percent nuclear rupture (Mean ± SE) in GFP and dMMP transfected cells. **(C)** Cell viability (Mean ± SE) in dMMP transfected cells normalized to GFP Control. Data are averaged across three independent experiments. Bars with * represents significant difference compared to control at *p* < 0.05.

To better understand the function of MMPs in neurons, we expressed GFP-dMMP constructs in SH-SY5Y cells using Lipofectamine 200 transection reagent. SH-SY5Y neuroblastomas are an excellent cell line for modeling neuronal pathways due to their expression of neuronal receptors and proteins and their ability to differentiate into a neuron-like phenotype (Hearst et al., 2011). However, we found the combination of GFP-dMMP plasmids and Lipofectamine to be highly toxic to the cells as compared to GFP plasmids and Lipofectamine (Figure 2). Lipofection has been reported to have some toxic effects in cultured cells (Zhong et al., 2008). Our results suggested that expression of dMMPs increased lipofection toxicity as compared to GFP alone. We saw a significant increase in nuclear rupture in cells expressing GFP-dMMPs as compared to GFP alone lipofection experiments (Figures 2A,B). We also saw significant reduction in cell viability in cells transfected with GFP-dMMPs as compared to the empty GFP vector (Figure 2C). Next, we assessed cell lysates via western blotting and found a significant increase in Casp-3 cleavage in cells lipofected with GFP-dMMPs as compared to the empty GFP vector (Supplementary Figures 5A,B). We found no significant difference between full length GFP-dMMPs compared to active GFP-dMMPΔPPs. Taken together, this data suggests a possible role for dMMPs in triggering apoptotic pathways resulting in reduced cell viability.

### dMMPs Expression in SH-SY5Y Cells Induces Neuronal-Like Phenotype

**FIGURE 3:**
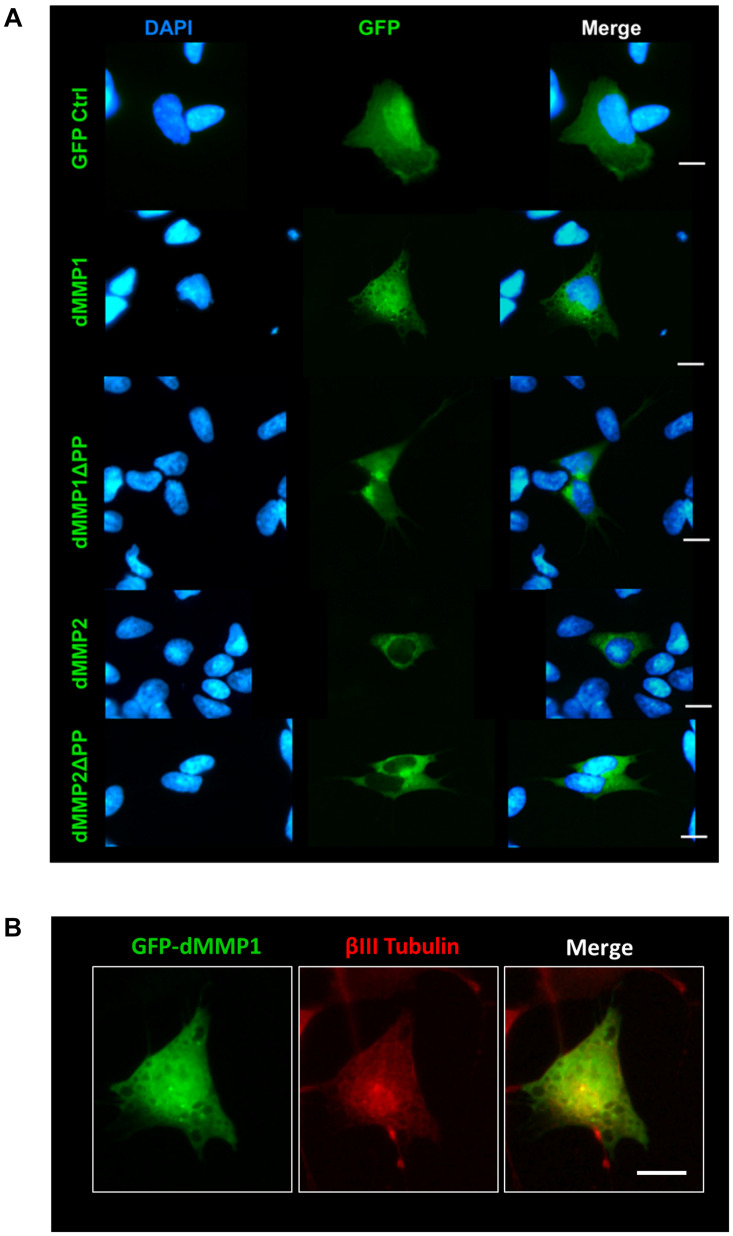
GFP-dMMP localization in neuronal cells. **(A)** Transfection of SH-SY5Y cells with GFP-dMMPs and GFP alone, using Fugene 6, and localization patterns of GFP at 24 h post-transfection. GFP Control localized to the nucleus and cytoplasm whereas GFP-dMMP constructs localized differentially in SH-SY5Y neuroblastoma cells. Shown in blue is DAPI nuclear stain. Shown in green are the various GFP constructs compared to GFP control. **(B)** An enlarged image of GFP-dMMP1 (green) co-localization with βIII Tubulin (red). Scale bar = 10 μm.

**FIGURE 4:**
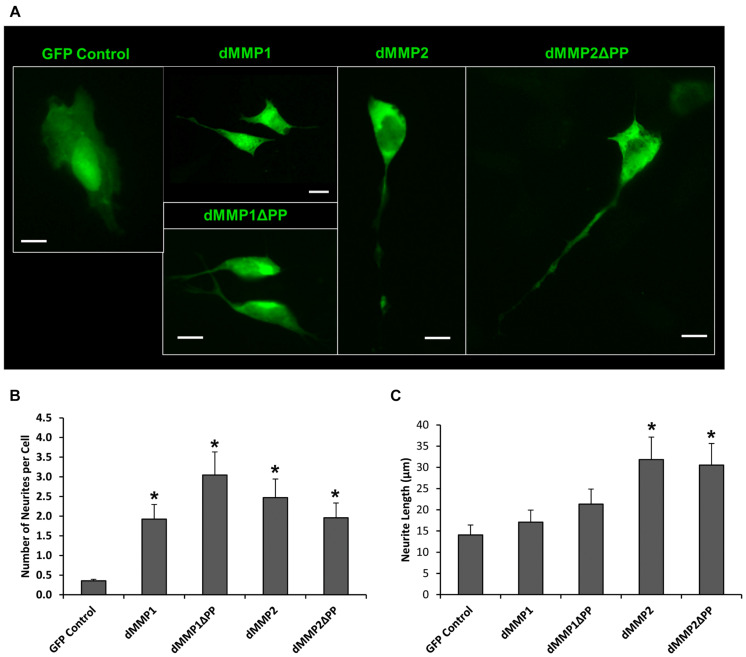
GFP-dMMP expression induces neurite formation. **(A)** Neuron like morphology of SH-SY5Y cells transfected with GFP Control and GFP-dMMP constructs. Scale bar = 10 μm. **(B)** Number of neurites per cell (Mean ± SE) in GFP Control and GFP-dMMP transfected cells. **(C)** Neurite length (μm) (Mean ± SE) in GFP Control and GFP-dMMP transfected cells. Data are averaged across three independent experiments. Bars with * represents significant difference compared to control at *p* < 0.05.

**FIGURE 5:**
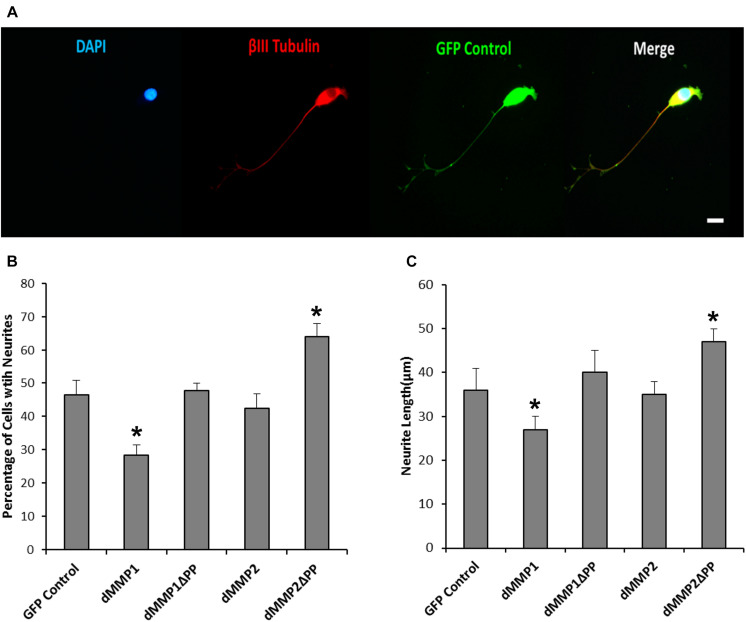
Differential dMMP expression impacts neural differentiation. **(A)** 12-*O*-tetradecanoylphorbol-13-acetate (TPA) differentiated stable SH-SY5Y cells expressing GFP. Shown in blue is DAPI nuclear stain, in red is βIII Tubulin, and green is GFP. Scale bar = 10 μm. **(B)** Percentage of cell with neurite (Mean ± SE) in GFP and GFP-dMMP stable cell lines. **(C)** Neurite length (μm) (Mean ± SE) in GFP Control and GFP-dMMP stable cell lines. Data are averaged across three independent experiments. Bars with * represents significant difference compared to control at *p* < 0.05.

Using a non-liposomal transfection reagent, Fugene 6, we were able to lower dMMP toxicity and proceeded with GFP-dMMP localization studies. We transfected SH-SY5Y cells with GFP-dMMPs and GFP alone, using Fugene 6, and visualized the GFP localization patterns under the microscope at 24 h post-transfection. GFP Control localized to the nucleus and cytoplasm (Figure 3A). GFP-dMMP1 and GFP-dMMP1ΔPP displayed strong localization to the nucleus and cytoplasm whereas GFP-dMMP2 and GFP-dMMP2ΔPP localized weakly to the nucleus and strongly to the cytoplasm (Figure 3A). We did not notice a difference between localization patterns in dMMP full length protein as compared to dMMPΔPP proteins with propeptide domain removed. dMMPs displayed a structural pattern at high magnification, so we stained slides for neuronal microtubule protein, βIII Tubulin. GFP-dMMPs co-localized with βIII Tubulin (Figure 3B). We also noticed a change in cellular morphology with the expression of GFP-dMMPs as compared to GFP alone. SH-SY5Y cells can be differentiated into neurons, changing their morphology, and production long neurites (Hearst et al., 2011). We visualized the SH-SY5Y cells expressing GFP or GFP-dMMPs at 48 h post transfection and saw a more neuronal-like-phenotype in cells expressing GFP-dMMPs as compared to GFP alone (Figure 4A). We saw a significant increase in the number of neurites per cell in cells expressing GFP-dMMPs and an increase neurite length in cells expressing GFP-dMMP2 constructs as compared to GFP alone (Figures 4B,C). Next, we expressed GFP and GFP-dMMPs in SH-SY5Y cell and created stable lines using G418 selection. We differentiated the stable lines into neurons using TPA treatment (Figure 5A). We saw differences in neurite formation and length based upon differing dMMP expression as compared to GFP alone. The TPA treated dMMP1 stable lines displayed a significant reduction in the percentage of cells with neurites and a reduction in neurite length as compared to GFP Controls (Figures 5B,C). The TPA treated dMMP2ΔPP stable lines displayed a significant increase in the percentage of cells with neurites and an increase in neurite length as compared to GFP Controls (Figures 5B,C). However, the TPA treated dMMP1ΔPP and dMMP2 stable lines displayed no significant difference in neurites percentages or lengths as compared to GFP Controls (Figures 5B,C). Taken together, this data suggests a possible role for dMMPs in neuronal differentiation.

### Inhibition of Endogenous MMPs Reduces Neuronal-Like Phenotype

**FIGURE 6:**
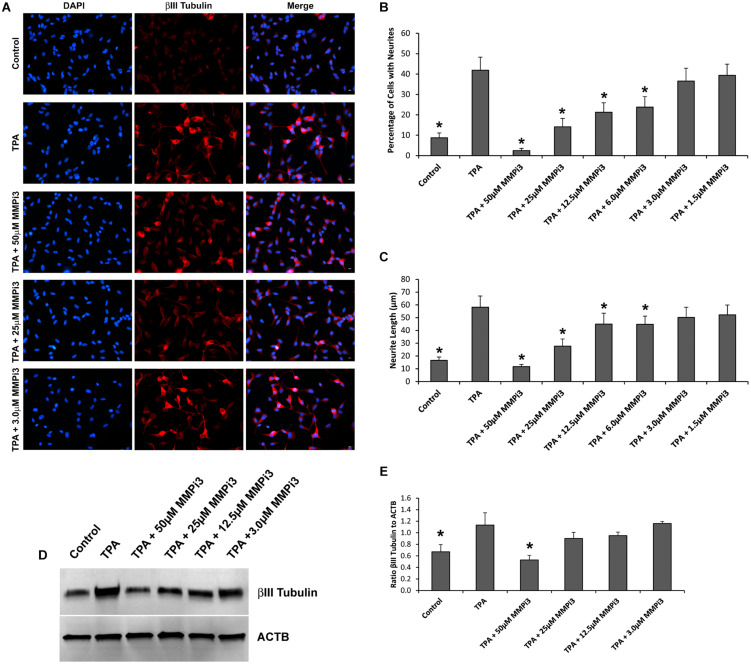
Matrix metalloproteinases inhibition impacts neural differentiation. **(A)** SH-SY5Y cells under control conditions, treated with TPA, or TPA plus various concentrations of MMP inhibitor III (MMPi3). Shown in blue is DAPI nuclear stain, in red is βIII Tubulin. Scale bar = 10 μm. **(B)** Percentage of cell with neurite (Mean ± SE) in Control, TPA treated, and TPA plus MMPi3. **(C)** Neurite length (μm) (Mean ± SE) in Control, TPA treated, and TPA plus MMPi3. **(D)** Western blots of βIII Tubulin and β-Actin (ACTB) levels in SH-SY5Y lysates from Control, TPA, and TPA plus MMPi3 treated cells. **(E)** Ratio (Mean ± SD) of βIII Tubulin and ACTB taken from optical density analysis of western blot data. Data are averaged across three independent experiments. * Represents significant difference compared to control at *p* < 0.05.

To explore the function of endogenous MMPs in neural differentiation, we treated SH-SY5Y cells with TPA and varying concentrations of MMP inhibitor III (MMPi3) and performed immunofluorescence and western blotting analysis of βIII Tubulin (Figure 6). MMPi3 (6–50 μM) significantly reduced TPA dependent neuronal-like phenotype in SH-SY5Y cells, and reduced the percentage of cells with neurites (Figures 6A,B) and neurite length (Figures 6A,C). MMPi3 at 50 μM also significantly reduced TPA dependent induction of the βIII Tubulin protein as compared to β-actin (ACTB) (Figures 6D,E). Taken together, this data suggests a possible role for endogenous MMPs in TPA mediated neuronal differentiation.

### dMMPs Expression in C6 Glial Cells Induces a Reactive Glial Phenotype

**FIGURE 7:**
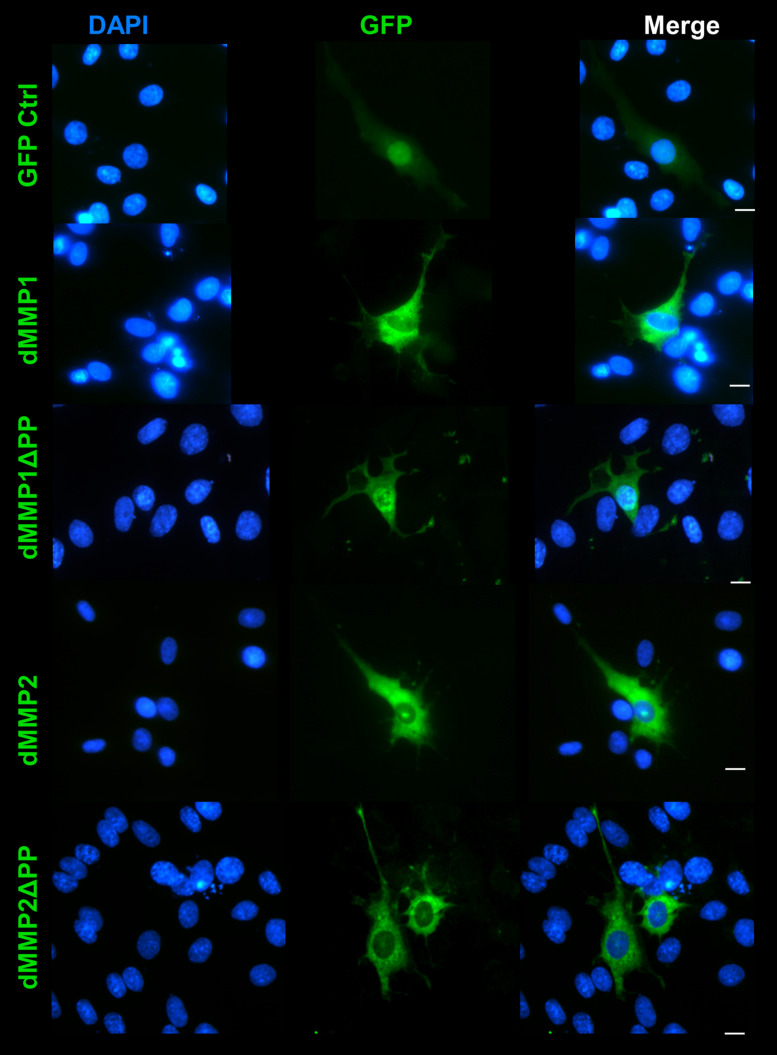
GFP-dMMP localization in glial cells. Transfection of C6 cells with GFP-dMMPs and GFP alone, using Fugene 6, and localization patterns of GFP at 24 hrs post-transfection. GFP Control localized to the nucleus and cytoplasm whereas GFP-dMMP constructs localized differentially in C6 glial cells. Shown in blue is DAPI nuclear stain. Shown in green are the various GFP constructs compared to GFP control. Scale bar = 10 μm.

**FIGURE 8:**
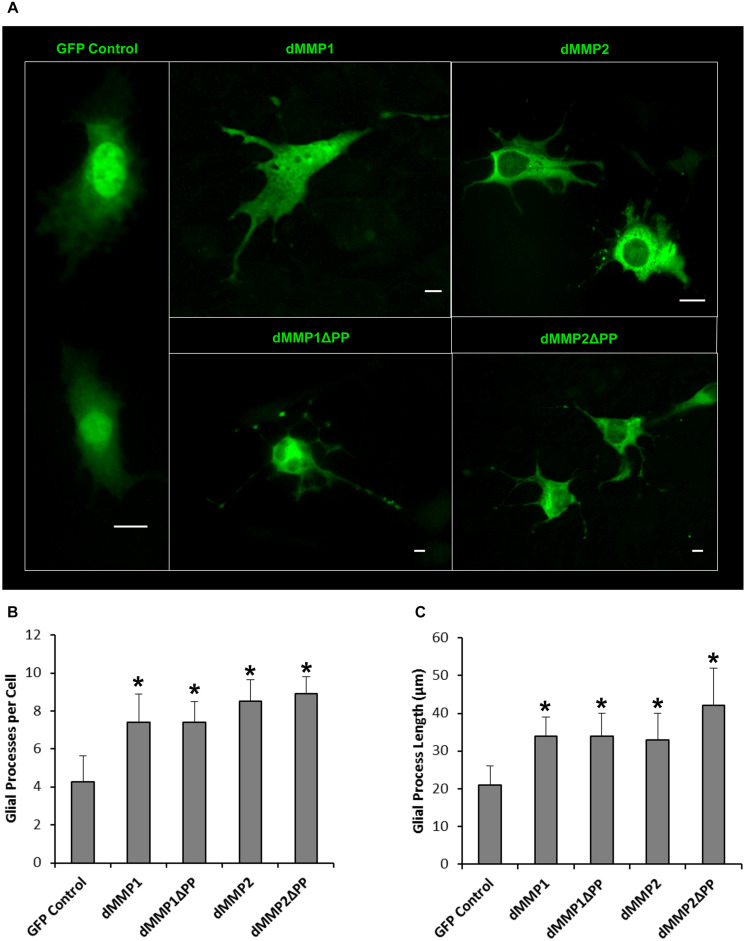
Expression of dMMPs induced glial process formation in C6 glioblastoma cells. **(A)** Glial-like morphology of C6 cells transfected with GFP Control and GFP-dMMP constructs. Some images are zoomed out to capture total cell size and shape. Each scale bar = 10 μm regardless of zoom. **(B)** Number of glial processes per cell (Mean ± SE) in GFP Control and GFP-dMMP transfected cells. **(C)** Glial process length (μm) (Mean ± SE) in GFP Control and GFP-dMMP transfected cells. Data are averaged across three independent experiments. Bars with * represents significant difference compared to control at *p* < 0.05.

The rat C6 glioblastomas are an excellent cell line for modeling glial pathways due to their expression of glial proteins and their ability to differentiate into an astrocyte-like phenotype (Ozawa et al., 2013; Chao et al., 2015). To better understand the function of MMPs in glia, we expressed GFP-dMMP and GFP Control constructs in C6 cells using Fugene 6 transfection reagent. We saw similar localization patterns as seen in the SH-SY5Y cells. GFP Control localized to the nucleus and cytoplasm (Figure 7). GFP-dMMP1 and GFP-dMMP1ΔPP displayed strong localized to the nucleus and cytoplasm whereas GFP-dMMP2 and GFP-dMMP2ΔPP localized weakly to the nucleus and strongly to the cytoplasm (Figure 7). We did not notice a difference between localization patterns in dMMP full length protein as compared to dMMPΔPP proteins with propeptide domain removed. However, we noticed a distinctive change in glial morphology in C6 cells expressing GFP-dMMPs as compared to GFP alone. We recorded a more reactive-glial-like phenotype or microglia-like phenotype in cells expressing GFP-dMMPs as compared to GFP alone at 48 h post-transfection (Figure 8A). We saw a significant increase in the number of glial processes per cell and an increase in glial process length in cells expressing GFP-dMMPs as compared to GFP alone (Figures 8B,C). This data suggests that dMMPs may play a role in glial morphological changes.

### Inhibition of Endogenous MMPs Induces Distinct Glial Morphologies

**FIGURE 9:**
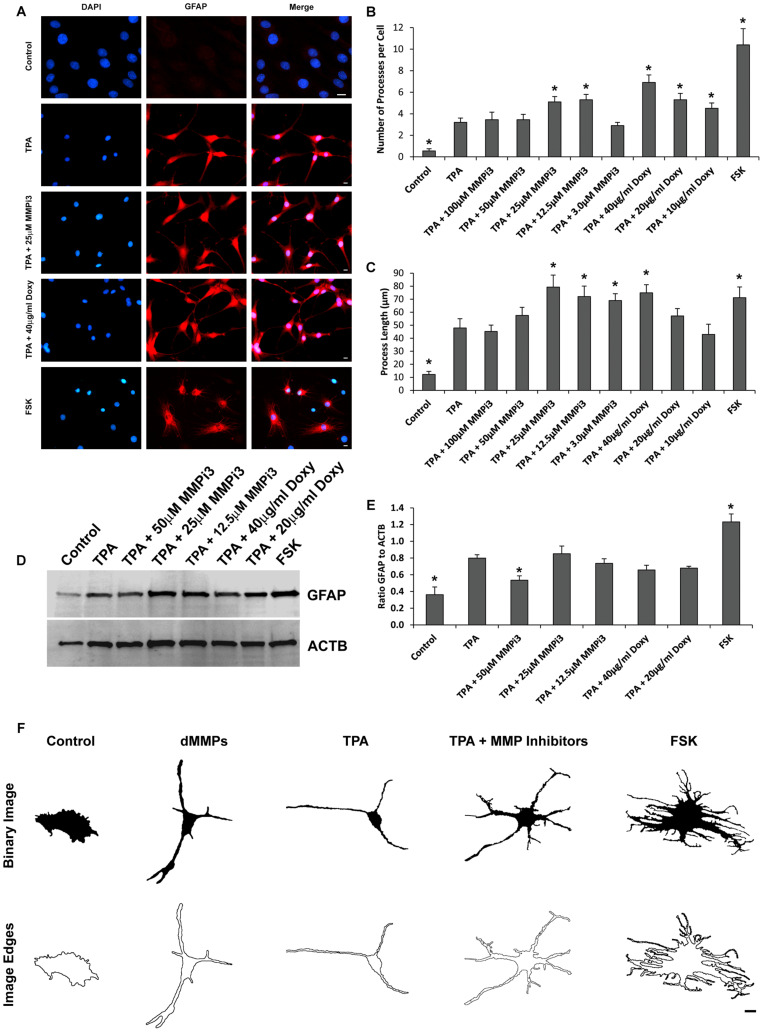
Matrix metalloproteinases inhibition impacts C6 glial cell morphology. **(A)** C6 cells under control conditions, treated with TPA, TPA plus various concentrations of MMP inhibitor III (MMPi3), TPA plus various concentrations MMP inhibitor doxycycline (Doxy), or MMP suppressor forskolin (FSK). Shown in blue is DAPI nuclear stain, in red is GFAP. Scale bar = 10 μm. **(B)** Number of processes per cell (Mean ± SE) in Control, TPA treated, TPA plus MMPi3, TPA plus Doxy, and FSK. **(C)** Process length (μm) (Mean ± SE) in Control, TPA treated, TPA plus MMPi3, TPA plus Doxy, and FSK. **(D)** Western blots of GFAP and β-Actin (ACTB) levels in C6 lysates from Control, TPA, TPA plus MMPi3, TPA plus Doxy, and FSK treated cells. **(E)** Ratio (Mean ± SD) of GFAP and ACTB taken from optical density analysis of western blot data. Data are averaged across three independent experiments. * Represents significant difference compared to control at *p* < 0.05. **(F)** Modulation of MMPs results in distinct C6 glial cell phenotypes. Shown are representative Binary Images and the Image Edges of a C6 cell expressing dMMPs, treated with TPA, TPA plus MMP inhibitors, or FSK. Scale bar = 10 μm.

**FIGURE 10:**
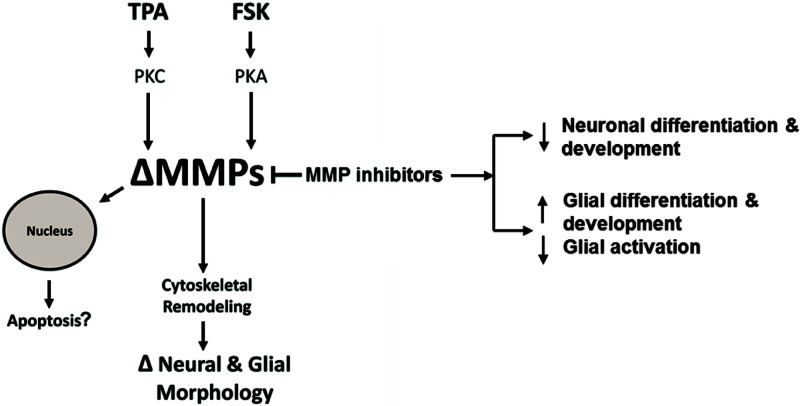
Hypothetical intracellular MMP Signaling Pathways. Hypothetical pathways that alter intracellular MMPs resulting in neural and glial cell morphology changes or apoptosis. TPA activates PKC signaling resulting in altered MMP activity or expression (ΔMMPs). FSK activates PKA signaling resulting in ΔMMPs. Intracellular ΔMMPs promote cytoskeletal remodeling, changing neural and glial cell morphology (ΔNeural & Glial Morphology). MMP inhibitors modulate MMP activity resulting in decreased neuronal growth and differentiation while increasing glial growth and differentiation. MMP inhibition, on the other hand, can inhibit glial activation. ΔMMPs can enhance MMP nuclear localization leading to possible apoptosis.

To further explore the role of endogenous MMPs in C6 cell differentiation, we treated cells with TPA and varying concentration of MMP inhibitors MMPi3 and doxycycline (Doxy) and performed immunofluorescence and western blotting analysis of glial fibrillary acidic protein (GFAP) (Figure 9). We also treated cells with forskolin (FSK), a modulator of MMP expression and assessed cell morphology and GFAP protein levels. TPA produced a more astrocyte-like-phenotype as compared to untreated C6 cells as revealed by GFAP staining (Figures 9A, 10). TPA treatment significantly increased the number of processes per cell and increased process length as compared to untreated controls (Figures 9B,C). Interestingly, MMPi3 treatment (12.5–25 μM) significantly enhanced the TPA dependent glial-like phenotype in C6 cells, and increased the number of processes per cell (Figures 9A,B) as well as process length (Figures 9A,C). Doxy treatment at 40 μg/ml also significantly enhanced the TPA dependent glial-like phenotype in C6 cells by increasing the number of processes per cell (Figures 9A,B) and increasing process length (Figures 9A,C). Treatment with FSK alone, induced another significant change in glial morphology, where C6 cells showed a significant increase in the number of processes per cell (Figures 9A,B) and an increase in process length (Figures 9A,C) as compared to TPA treatment alone. TPA induced GFAP protein expression was significantly decreased by treatment with 50 μM MMPi3; however, Doxy had little effect on TPA induced GFAP protein expression (Figures 9D,E). FSK treatment alone significantly increased GFAP protein expression as compared to TPA treatment (Figures 9D,E). Interestingly, the glial phenotypes between TPA treatment, dMMP expression, MMP inhibition, and FSK treatment were all quite distinct in appearance (Figure 9F). To stress this point further, we converted representative images of a C6 cell under the various conditions into a Binary Image and then found the edges to create an Edged Image (Figure 9F). dMMP expressing cells had a phagocytic glia phenotype, TPA treatment resembles more of an astrocytic phenotype, TPA + MMP inhibitors resembled more of a resting glial phenotype, and FSK induced a reactive glial phenotype (Figure 9F). Taken together, this data suggest MMPs may play an important role in glial differentiation, morphology changes, and glial activation.

## Discussion

For successful establishment of a functional nervous system, it is essential that MMP activity be coordinated both spatially and temporally. However, due to the complex nature of MMP families, efforts to resolve the molecular mechanisms and linking individual MMPs to their substrates *in vivo* has been significantly hindered. To circumvent these challenges, it is essential to utilize model organisms with genomes encoding MMP families most suited to the research question being asked. This entails balancing simplicity and experimental tractability versus redundancies of the MMP family and translatability to human biology. While MMPs are central components of the molecular mechanisms underlying neural cell fate, morphogenesis, migration, homeostasis, function and pathology; these mechanisms entail not only their traditional functions in ECM remodeling, but also roles in modulating cell–cell adhesion, signal transduction and perhaps poorly understood intracellular activities. MMPs’ protein domains are conserved across eukaryotic species. These functional domains include: the catalytic domain that is zinc-dependent, the zymogen autoinhibitory pro-domain, and a hemopexin domain for substrate recognition (Nagase et al., 2006). Some MMPs tether to the cellular membrane via a transmembrane domain or a GPI anchor, while other MMPs are secreted (Nagase et al., 2006). In Drosophila, both dMMP1 and dMMP2 have secreted as well as membrane tethered forms (LaFever et al., 2017). Both dMMPs are thought to have distinct functions due to their substrate specificity. The protein sequence homology study between dMMP1 and dMMP2 and hMMPs shows that while sequence identity varied between 34 and 40%, all the catalytic active sites were identical. This homology data suggests that homologs of dMMPs in humans may have similar cellular function and may even target similar substrates. Moreover, NLS were predicted in both dMMP1 (three sequences) and dMMP2 (a single sequence). This suggests that dMMPs have the ability to translocate to the nucleus.

Since we found homology between dMMPs and some hMMPs, we suggest that the analysis of dMMPs’ intracellular function may provide insight into the function of human MMP homologs in the CNS. Expanding on this idea, we expressed GFP tagged dMMPs in neuronal and glial cell lines. We found a correlation between lipofection and dMMP toxicity. Lipofection increased dMMP toxicity as compared to GFP Control, increasing nuclear rupture, reducing cell viability by possibly inducing apoptosis. Lipofection has been shown to cause apoptosis in certain cell types (Zhong et al., 2008). MMPs have been suggested to have multiple extracellular, intracellular, and intranuclear functions in programmed cell death (PCD) pathways (Mannello et al., 2005). While excessive PCD can be harmful and can contribute to various degenerative pathologies, a lack of PCD can contribute to proliferative disorders (Green and Evan, 2002). Apoptosis is one type of PCD that is mainly dependent on caspase proteases (Cryns and Yuan, 1998). In this study we found that differential dMMP expression promotes an increase of cleaved Caspase-3 levels in neuronal cells triggering apoptosis. MMPs have been implicated in apoptosis, where nuclear localization of GFP-MMP3 enhances cell death in CHO cells (Si-Tayeb et al., 2006). MMP2 have been shown to localize to the nucleus of cardiomyocytes resulting in PARP cleavage (Kwan et al., 2004). MMPs were able to directly cleave and activate effector caspases by baculovirus encoded fibroblast growth factors (vFGF) during infection of insects (Means and Passarelli, 2010). Hence, our data suggests that dMMPs and their human homologs may have intracellular function in CNS apoptotic pathways.

To further explore the intracellular function of dMMPs, we switched to a non-liposomal transfection method. GFP-dMMP1 constructs localized to the cytoplasm and nucleus in neural and glial cell lines, while GFP-dMMP2 constructs localized to the cytoplasm. We did not see a difference between localization patterns in full length GFP-dMMPs compared to active dMMPΔPP proteins, where the propeptide domain is removed. These results suggest that the N-terminal GFP tag may have been hindering the function of the propeptide domain or that the active and the full length dMMPs have similar localization. A similar study was performed using human MMP3 with a GFP tag and revealed intracellular function of MMPs, where GFP-MMP3 expression resulted in localization to the nucleus and cytoplasm in CHO cell lines (Si-Tayeb et al., 2006). The intracellular function of MMPs was further supported by the dramatic changes in cell morphology induced by expression of dMMPs in neural and glial cell lines. SH-SY5Y cells displayed a more neuron-like-phenotype when expressing GFP-dMMPs as compared to GFP, where dMMPs increased neurite formation and neurite length. SH-SY5Y stable lines expressing dMMPs indicate possible distinct roles for dMMP1 and dMMP2 in neural cell differentiation; however, we also advocate caution in the use of stable lines as the best approach for studying MMPs *per se*. We saw similar phenotypes in TPA treated SH-SY5Y cells as compared to cells expressing dMMPs, which can confound the results and make it difficult to attribute MMPs with a role specifically when using stable cell lines. TPA has been shown to increase MMP9 expression in SH-SY5Y cells (Farina et al., 1999). To explore the intracellular function of MMPs further, we targeted endogenous MMPs in SH-SY5Y cells using different concentrations of the MMP inhibitor MMPi3 plus TPA. Inhibiting MMPs with MMPi3, significantly reduced TPA dependent neuronal-like phenotype and significantly reduced TPA dependent induction of the βIII Tubulin protein. Taken together, this data suggests a possible role for MMPs in neuronal differentiation and morphological changes such as neurite development.

Expression of MMPs in C6 glioblastoma had a dramatic effect on glial cell morphology, where dMMPs increased the number of glial processes per cell as well as increased process length. Differentiation of C6 cells with TPA resulted in a similar phenomenon; however, TPA treated cells most resembled astrocytes, while dMMP expressing cells resembled microglia or reactive glial. This comes as no surprise, since up-regulation of MMPs is associated with a neuroinflammation, macrophage activation, and glial activation (Yin et al., 2006; Rosenberg, 2009; Brkic et al., 2015a; Rivera et al., 2019). When microglia become reactive, they can cause severe damage to neurons, the ECM of the CNS, and the BBB by excessive production of cytokines and MMPs (Yin et al., 2006; Rosenberg, 2009; Brkic et al., 2015a; Rivera et al., 2019). Since some MMPs can cleave the propeptide domain of other MMPs, excess MMP production would in turn activate more MMPs influencing cell morphology. It is possible that activation of endogenous MMPs by dMMPs is altering glial morphology. To further explore the importance of MMPs in glial morphology, we treated C6 cells with PKC activator, TPA, and inhibited endogenous MMPs with MMPi3 and Doxy. Interestingly, both MMPi3 and Doxy enhanced TPA induced glial morphology changes. Both MMP inhibitors increased glial processes per cell and process length. MMPi3 at 50 μM caused a reduction in GFAP protein expression as compared to TPA alone. To go one step further, we treated C6 cells with PKA activator, FSK, a modulator of MMP expression. FSK has been shown to suppress MMP1 and MMP9 expression and increase TIMP1 expression (Ben-Shlomo et al., 2003; Park et al., 2010). FSK treatment increased the number of processes per cell, increased process length and enhanced GFAP protein expression. The various treatment conditions resulted in distinct glial phenotypes, suggesting that intracellular MMPs may function in glial differentiation and glial activation pathways.

Based on our study, a functional role of MPPs in cell morphology and internal structure is starting to come to light. We propose a hypothetical pathway to explain the changes in neural and glia cellular morphology or apoptosis due to altered intracellular MMP expression or activity (Figure 10). According to this model, TPA can activate PKC signaling resulting in altered MMP activity or expression (ΔMMPs). FSK, in turn, can activate PKA signaling resulting in ΔMMPs. Intracellular ΔMMPs promote cytoskeletal remodeling, changing neural and glial cell morphology. MMP inhibitors modulate MMP activity resulting in decreased neuronal differentiation and development whereas in glial cells it increases glial differentiation and development, while inhibiting glial activation. Altered MMP expression or activity can enhance MMP nuclear localization leading to possible apoptosis.

We saw co-localization of GFP-dMMPs with structural protein βIII Tubulin. This data alongside the significant morphological changes induced by MMP expression suggests a functional role of MMPs in cellular structure. Previous studies have shown that the expression of MMPs is linked to structural changes in many different cell types: MMP2 activation impacts actin cytoskeletal organization in HTM cells resulting in significant morphological changes (Sanka et al., 2007), alterations in cell shape in melanoma cells have been linked to MMP9 suppression (MacDougall and Kerbel, 1995), contractility of cultured cardiomyocytes is associated with MMP2 expression (Bildyug et al., 2015; Bildyug, 2016). MMPs have been shown to degrade a wide range of structural proteins such as actin, myosin, troponin, tinin and as well as mitochondrial proteins just to name a few (Wang et al., 2002; Sawicki et al., 2005; Sung et al., 2007; Moshal et al., 2008; Butler and Overall, 2009; Ali et al., 2010). Strong evidence for the intracellular function of MMPs comes from reports that indicate MMPs are required for rapid reorganization of the actin cystokeleton and the remodeling of focal adhesion in MSC cells (Meriane et al., 2006). Internal MMPs mediated signaling events that modulate cytoskeletal remodeling via RhoA/ROCK and MEK1/ERK intracellular signaling pathways (Meriane et al., 2006). This mechanism may explain the drastic changes in neural and glial morphology seen upon dMMP expression. In agreement to other studies our results suggest that MMPs play significant intracellular roles in apoptosis and cytoskeleton remodeling in neurons. Moreover, our data suggests intracellular MMPs may contribute to glial activation warranting further research to elucidate the role of MMPs in CNS disorders. In conclusion, our robust *in vitro* model adds a powerful experimental toolkit to elucidate the precise molecular mechanisms by which MMPs exert their intracellular actions while also providing an evolutionary perspective on the fundamental mechanisms that underlie the formation and function of MMPs in animal nervous systems.

## Data Availability Statement

The original contributions presented in the study are included in the article/Supplementary Material, further inquiries can be directed to the corresponding author/s.

## Author Contributions

SH and NK were involved in conceiving and designing the work. SH, AB, BD, KJ, DM, CT, JS, EK, JW, and NK were involved in conducting various aspects of the work including transfection experiments, acquiring images, quantifying data, and western blotting. SH, AB, EK, JW, and NK were involved in data curation, generating graphs, and statistical analysis. SH, AB, and NK were involved in writing and editing the manuscript. All authors approved the final version of the manuscript for publication.

## Conflict of Interest

The authors declare that the research was conducted in the absence of any commercial or financial relationships that could be construed as a potential conflict of interest.

## Footnotes

1 http://www.clustal.org/omega/

2 http://tcoffee.crg.cat/apps/tcoffee/index.html

3 https://embnet.vital-it.ch/software/BOX_form.html

4 http://nls-mapper.iab.keio.ac.jp/cgi-bin/NLS_Mapper_form.cgi

5 https://imagej.nih.gov/ij/
