# Supplementary material for: Expression of Drosophila Matrix Metalloproteinases in Cultured Cell Lines Alters Neural and Glial Cell Morphology
Source: Front Cell Dev Biol. 2021 May 13;9:610887. doi: 10.3389/fcell.2021.610887 (PMC8155609; doi:10.3389/fcell.2021.610887)
Supplement: Supplementary file 1 [file Data_Sheet_1.PDF]

Supplementary Material

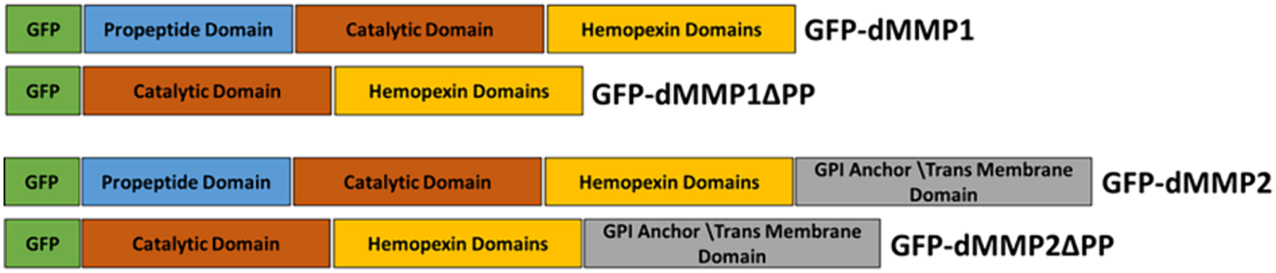

Supplemental Figure S1: Schematic diagrammatic representation of GFP-dMMP plasmid construct designs for dMMP used for expression in cell culture models. ΔPP constructs denote the removal of the propeptide domain to express the active dMMP. GFP domain is shown in green, the propeptide domain in blue, the catalytic domain in orange, the hemopexin domain in yellow and the GPI Anchor\Transmembrane domain in grey.

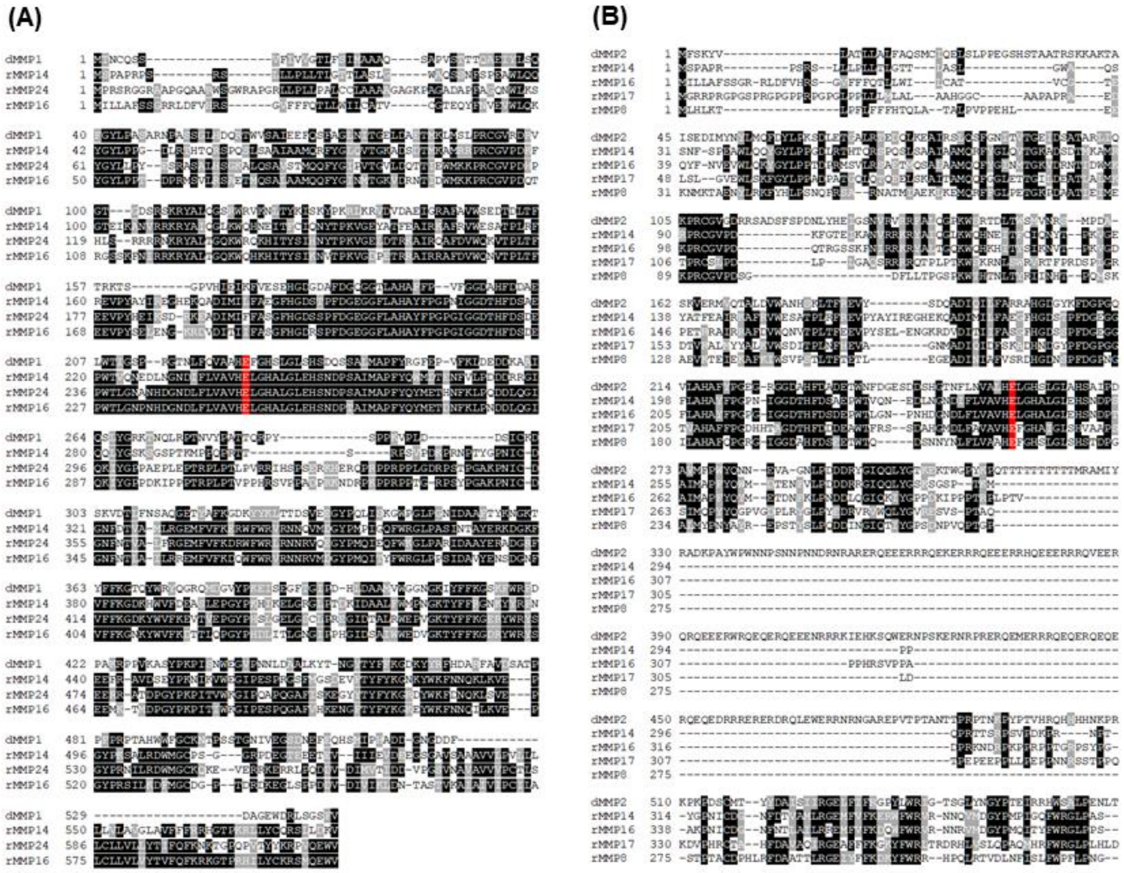

Supplementary Figure S2: Amino acid sequence alignments of (**A**) dMMP1 and (**B**) dMMP2 with *Rattus norwegicus* homologues. Identical amino acids are shown in black boxes and similar amino acids are highlighted in grey boxes. Gaps have been introduced to permit alignment. Conserved active sites are shaded in red. Uniprot entries: *Drosophila melanogaster* dMMP1 (Q8MLN6) showed alignment to rMMP14 (Q10739), rMMP16 (F1M7F5) and rMMP24 (Q99PW6) whereas dMMP2 (Q8MPP3) showed alignment to rMMP8 (G3V7D0), rMMP14 (Q10739) and rMMP16 (F1M7F5).

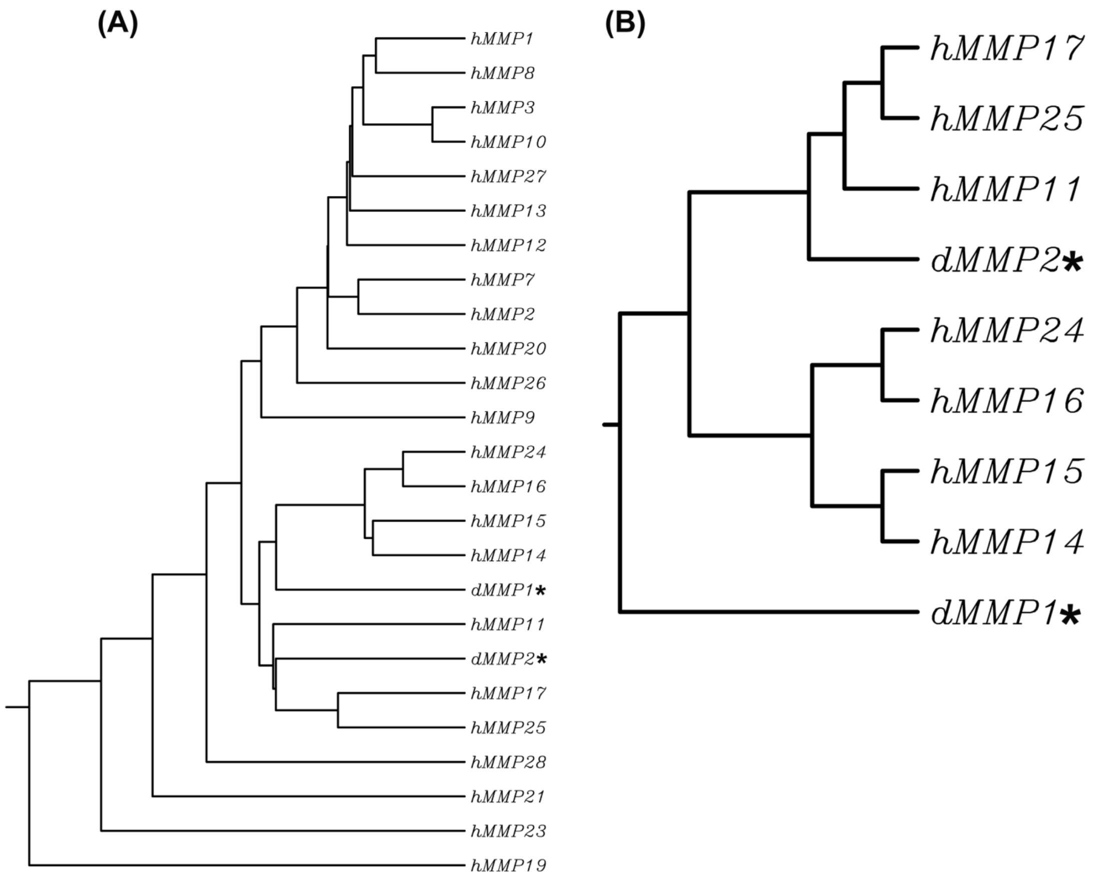

Supplementary Figure S3. Phylogenetic relationships of dMMP1 and dMMP2 proteins from *Drosophila melanogaster* with human MMPs. The neighbor-joining tree was generated in MEGA5 with 1000 bootstrapping. dMMPs are denoted with an * mark. (**A**) Relationship with dMMP1 and dMMP2 with 23 human MMPs (**B**)The sequences for alignment and phylogenetic tree were obtained from the following Uniprot entries: *Drosophila melanogaster* dMMP1 (Q8MLN6) and dMMP2 (Q8MPP3); *Homo sapiens* MMPs were hMMP11 (B3KQS8), hMMP14 (P50281), hMMP15 (P51511), hMMP16 (P51512), hMMP17 (Q9ULZ9), hMMP24 (Q9Y5R2) and hMMP25 (Q9NPA2).

(A)

Predicted bipartite NLS		
**Pos.**	**Sequence**	**Score**
98	RVGTGDSRSKRYALQGSRWRVKNLTYKISKYPKR	6.1
103	DSRSKRYALQGSRWRVKNLTYKISKYPKR	7.9
425	RPPVKASYPKPISNWEGVPNNLDAALKYTNGY	5.3

(B)

Predicted bipartite NLS		
**Pos.**	**Sequence**	**Score**
107	RCGVGDRRSADSFSPDNLYHEIGSNVRVRRFAL	4.1

Supplementary Figure S4. Nuclear localization signal (NLS) sequences were predicted using NLS mapper. It revealed that **(A)** dMMP1 has three putative sequences that signal it to localize in the nucleus whereas (B) dMMP2 had a single NLS sequence.

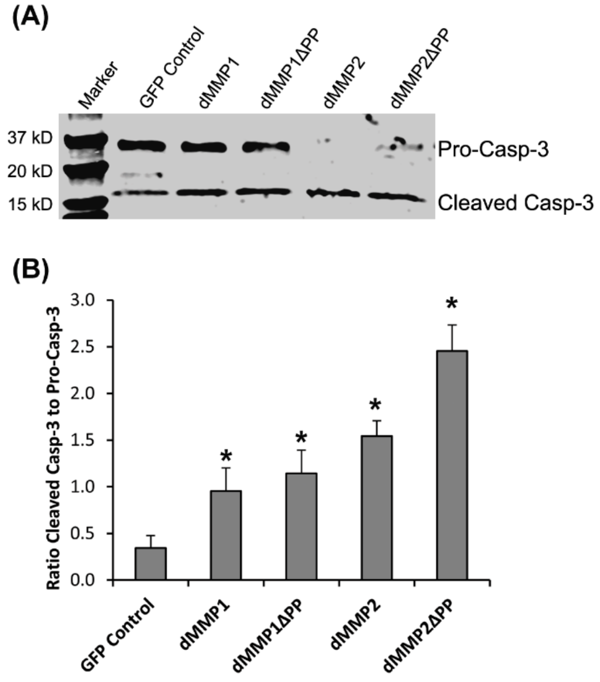

Supplementary Figure S5: dMMP expression may trigger apoptosis in neuronal cells (A) Western blots of Pro-Casp-3 and Cleaved Casp-3 levels in cell lysates at 48 hrs post lipofection with GFP and dMMP constructs. **(B)** Ratio (Mean ± SD) of Cleaved Casp-3 to Pro-Casp-3 taken from optical density analysis of western blot data. Data are averaged across three independent experiments. Bars with * represents significant difference compared to control at $p<0.05$.
